# Giant Cell Arteritis with Concomitant Chronic Myelomonocytic Leukemia

**DOI:** 10.4274/tjh.galenos.2020.2020.0357

**Published:** 2021-08-25

**Authors:** Mert Öztaş, Ilkın Muradov, Abdülkadir Erçalışkan, Ahu Senem Demiröz, Şebnem Batur, Ahmet Emre Eşkazan, Serdal Uğurlu

**Affiliations:** 1 İstanbul University-Cerrahpaşa, Cerrahpaşa Faculty of Medicine, Department of Rheumatology, İstanbul, Turkey; 2 İstanbul University-Cerrahpaşa, Cerrahpaşa Faculty of Medicine, Department of Internal Medicine, İstanbul, Turkey; 3 İstanbul University-Cerrahpaşa, Cerrahpaşa Faculty of Medicine, Department of Hematology, İstanbul, Turkey; 4 İstanbul University-Cerrahpaşa, Cerrahpaşa Faculty of Medicine, Department of Pathology, İstanbul, Turkey

**Keywords:** Giant cell arteritis, Vasculitis, Chronic myelomonocytic leukemia, Leukemia

A 62-year-old man with no previous medical conditions was hospitalized with constitutional symptoms, which had started within the past 2 months. He described recurrent fever (>39 °C), weight loss, and headache. Initial physical examination was unremarkable except for pallor of conjunctiva and fever (39.3 °C). His hemoglobin was 7.5 g/dL (normal range: 13-17.2 g/dL), while white blood cell (WBC) and platelet counts were 22.8x10^9^/L (normal: 4.3-10.3x10^9^/L) and 58.3x10^9^/L (normal: 150-400x10^9^/L), respectively. He also had remarkable monocytosis of 6.2x10^9^/L (normal: 0.3-0.9x10^9^/L). He had elevated C-reactive protein of 150 mg/L (normal: <5 mg/L) and an erythrocyte sedimentation rate of 140 mm/h. His blood and urine cultures were sterile. On the suspicion of vasculitis and in order to exclude concomitant malignancy, positron emission tomography-computed tomography (PET-CT) was ordered, which showed both aortic arch and ascending aorta involvement without any signs of solid tumors (Figure 1). Diffuse bone marrow fluorodeoxyglucose uptake was also observed on PET-CT. Fragmentation of the lamina elastica interna was detected upon temporal artery biopsy without any sign of active vasculitis. He was diagnosed with giant cell arteritis (GCA) based on his age, initial erythrocyte sedimentation level, headache, fever, and large vessel involvement in PET-CT. He was further diagnosed with chronic myelomonocytic leukemia (CMML) with peripheral blood monocytosis (≥1x10^9^/L and monocytes accounting for ≥10% of the WBC differential count) [1]. Peripheral blood smear revealed 8% blasts and bone marrow aspiration and biopsy were consistent with CMML-1 (Figure 2). Prednisolone was initiated at 40 mg/daily and his constitutional complaints were resolved. Azacytidine was planned to be initiated for CMML, but the patient decided to receive this treatment in his hometown.

While the pathogenic link between GCA and CMML has not been clearly established so far, an association is commonly observed between GCA and myelodysplastic syndromes [2].

## Figures and Tables

**Figure 1:**
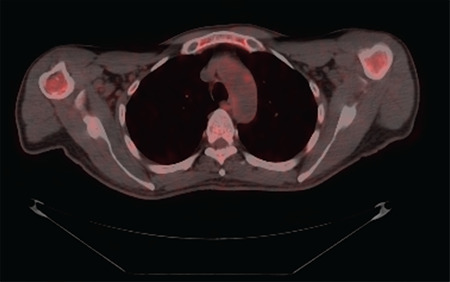
PET-CT showed aortic arch involvement and diffuse bone marrow FDG uptake. PET-CT: Positron emission tomography-computed tomography, FDG: fluorodeoxyglucose

**Figure 2:**
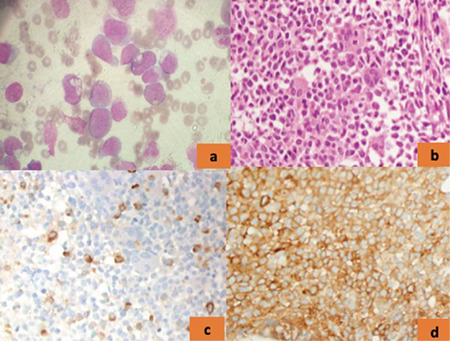
a) Bone marrow aspiration showed monocytic cells of over 20% and monoblasts of 7%, with dysplasia in all cell lineages (Giemsa 1000^x^). b) Bone marrow biopsy showed hyperplasia and dysplasia in all cell lineages (hematoxylin & eosin, 400^x^). c) Immunohistochemically, MPO is positive in very few cells (400^x^). d) CD33 showed that monocytic cells are increased (400^x^).

## References

[1] Arber DA, Orazi A, Hasserjian R, Thiele J, Borowitz MJ, Le Beau MM, Bloomfield CD, Cazzola M, Vardiman JW (2016). The 2016 revision to the World Health Organization classification of myeloid neoplasms and acute leukemia. Blood.

[2] Roupie AL, de Boysson H, Thietart S, Carrat F, Seguier J, Terriou L, Versini M, Queyrel V, Groh M, Benhamou Y, Maurier F, Decaux O, d’Aveni M, Rossignol J, Galland J, Solary E, Willems L, Schleinitz N, Ades L, Dellal A, Samson M, Aouba A, Fenaux P, Fain O, Mekinian A;, on behalf of MINHEMON (French Network of Dysimmune Disorders Associated with Hemopathies) (2020). Giant-cell arteritis associated with myelodysplastic syndrome: French multicenter case control study and literature review. Autoimmun Rev.

